# Relationship between web-based illness scripts and the performance of medical students in orthopedic surgery placements

**DOI:** 10.5116/ijme.6135.d424

**Published:** 2021-09-30

**Authors:** Chirathit Anusitviwat, Theerawit Hongnaparak, Varah Yuenyongviwat, Khanin Iamthanaporn, Pakjai Tuntarattanapong, Jongdee Bvonpanttarananon, Sitthiphong Suwannaphisit

**Affiliations:** 1 Department of Orthopedics, Faculty of Medicine, Prince of Songkla University, Thailand

**Keywords:** Web-based, orthopedic, examination, illness script, performance

## Abstract

**Objectives:**

We aimed to explore the relationship between web-based orthopedic illness scripts and medical students' performance as assessed through examination results.

**Methods:**

This was a retrospective cohort study with 83 fifth-year medical students in an academic hospital. During a one-month placement, they were instructed to do web-based illness scripts. Their performances were assessed by examination in the last week. All recorded data about illness scripts and examination results were retrieved. The students were separated into high and low response groups based on completed illness scripts. The characteristics of the students between the two groups were compared. Pearson correlation coefficients and regression analysis were used to identify the relationship between illness scripts and examination results.

**Results:**

There were 56 students in the high-response and 27 in the low-response groups. The characteristics and examination scores were not significantly different between the groups, while there was a significant difference in script completion (t_(27)_=13.72, p<0.001). Using Pearson correlation, we found weak correlations without significance between completed scripts, illness script scores, and examination scores. We found no relationship between illness script scores and examination scores, even in the high response group, by regression analysis.

**Conclusions:**

The use of web-based orthopedic illness scripts did not correlate to the examination performance of medical students. A high number of scripts without variety and limited time for practicing may have obscured potential positive relationships. Illness scripts should be adjusted as appropriate for each school before being assigned. A further multi-center, prospective study is suggested to identify the correlations and investigate the influencing factors.

## Introduction

Several educational strategies are used to promote medical knowledge and clinical reasoning to enhance students' skills in making an accurate diagnosis.^1^ One of these strategies used for teaching in the clinical setting is illness scripts.^2^ The illness script is based on the script theory, which proposes a high-level conceptual knowledge structure kept in long-term memory, which can be retrieved from the storage to integrate the patients' information into an appropriate clinical context.^2^^-^^4^The illness script has three main components, including epidemiological factors or enabling conditions, pathophysiological processes, and the consequences such as signs and symptoms of the disease.^3^^,^^5^ Management can also be included in an 'extended' illness script.^6^^,^^7^ The concept of illness scripts is to provide a cognitive framework, rather than pure memorization, for diseases and help the student to organize knowledge structures to interconnect information in the memory to apply to either an examination or real-life patients.^4^^,^^5^^,^^8^ In other words, these scripts are designed to help guide the medical students to link various kinds of knowledge in their pre-clinical years and in clinical settings. The goal of these scripts is to assist medical students to form a disease framework and to facilitate diagnostic problem-solving.^9^^,^^10^

 After repeated practicing with the illness scripts, the medical student should be able to recognize a pattern, discard unnecessary info, and provide a diagnosis from the given information.^6^^,^^10^^,^^11^ In case scenario problems, the medical student should be able to distinguish relevant from irrelevant information from a case presentation and then gather the vital features from each main component of the scripts, such as peak incidence of the disease in adult/elderly patients (epidemiological factors), cause of disease from degenerative change/infection, or characteristics of pain (signs and symptoms of the disease).^12^ To distil this information into a differential diagnosis and identify the most likely diagnosis, they would integrate these screened clinical details with their stored knowledge. Developing a list of possible diseases and practicing diagnostic skills, leading to further appropriate investigation and management, contribute to their future medical expertise.^13^

 Various traditional script-based teaching methods, including problem-based learning, concept mapping, and self-explanations, have been used with medical students.2 However, nowadays, medical education is being gradually converted from traditional teaching to electronic or online learning.^14^ Web-based illness scripts as a part of online learning can be beneficial in providing diagnostic experiences to solve actual clinical problems.^9^ The advantage of online learning is that it can provide students a variety of experiences leading to a greater quantity of knowledge.^15^ Many factors may influence online learning success or failure, such as cultural resistance and already overworked teachers or students. ^16^^-^^18^ Shifting to an entire online learning medical curriculum is challenging and must be done carefully with appropriate and timely feedback loops to ensure a successful transition.^14^ Creating additional online educational courses containing illness scripts apart from traditional education may be preferable. Therefore, in our institution, we generated a series of case-based illness scripts as part of the online course for our students to increase their performance and integrate pre-clinical knowledge of orthopedics into a clinical setting.

This study aimed to determine whether web-based illness scripts correlated with the performance of fifth-year medical students in orthopedic surgery placements. We hypothesized that the illness scripts would be positively correlated with the students' performance. The results from this study will help improve the adaptation and improvement of illness scripts in future online orthopedic courses.

## Methods

### Study design and participants

We conducted a retrospective study of fifth-year medical students studying at the Orthopedics Department of the Faculty of Medicine at Prince of Songkla University in Thailand, following their rehabilitation and orthopedic surgery placements in the academic year of 2020. We enrolled all medical students who took part in the online educational course, except students who missed the examination in the last week of their rotation or failed the overall academic year. This resulted in 83 students in the study. All of them were Thai and had never failed any summative assessments. This study was approved by the Institutional Review Board, Faculty of Medicine, Songklanagarind Hospital, Prince of Songkla University.

### Illness scripts and student performance

In our curriculum, the fifth-year medical students are required to attend an orthopedic surgery placement for one month to gain experience in orthopedics. Due to the short orthopedics and rehabilitation rotation period, we have developed an additional online educational course in the learning management system (LMS). This online course provides learning resources, learning assignments, electronic lectures, online quizzes, and illness scripts. On the first day of the rotation, the fifth-year medical students are instructed to do as many optional assignments as possible, including illness scripts.

The web-based illness script exercises, especially for the fifth-year medical students, were generated by a group of orthopedic surgeons with more than five years of experience in undergraduate medical education and prepared for fifth-year medical students only. These special illness scripts were all web-based, case-based practical problems, covering the ten necessary domains the fifth-year students needed to learn. The illness scripts included 8 cases of elbow and hand problems, 10 cases of shoulder problems, 10 cases of foot and ankle problems, 8 cases of hip problems, 12 cases of knee problems, 12 cases of spine problems, 6 cases of pediatric orthopedic problems, 3 cases of orthopedic oncology problems, and 5 cases of orthopedic trauma problems. Each case scenario began with a stem that described the clinical presentation of a patient followed by two sections of questions, beginning with a multiple-choice question (MCQ) for diagnosis and followed by four short-answer questions linked to the first question with further exploration of signs and symptoms, epidemiology, and pathophysiology of the disease. The students could do any individual script only one time during their one-month placement. After they finished and submitted their answers, the correct answers were displayed, which they could review at any time. The completed illness scripts were registered after they answered the questions, each script had a total of five questions, and one point was awarded for each correct answer: one point per question with a total possible score of 5 points per script. The number of completed scripts was automatically recorded in our database. Only approved teachers were able to access the database.

In the last week of the rotation, each student's performance was assessed by examination to ensure they had achieved the learning objectives. Our summative assessment comprised practical and theoretical examinations evaluated by objective structured clinical examination and MCQs, respectively. We selected only the MCQ scores for data analysis as our MCQs are mostly comprehensive questions asking about possible diagnoses, consistent with the illness scripts questions, and do not rely on any subjective differences among the examiners. Every month, 40 questions were randomly selected from a question bank containing 200 questions. All MCQs were in the English language and began with a stem describing a particular clinical problem followed by five options, from which there was one best answer. The students needed to choose the correct answer to get a score. The examination results were recorded in both paper and electronic documents.

### Data collection

The demographic data of the included medical students were obtained from the Faculty of Medicine database with permission. We recorded these data in electronic documents, including gender, race, marital status, and cumulative grade point average (GPA), calculated by dividing total grade points from all academic years by the number of total credit hours.

We retrieved one-year data from our LMS database. The completed scripts and scores of each case scenario of the illness scripts from the included medical students were collected at the end of the 2020 academic year. The total number of completed scripts of each medical student was calculated and recorded individually. The scores from the web-based illness scripts in each of the ten orthopedic problem domains were summarized and recorded electronically. Additionally, we asked for permission from the undergraduate education division of the Orthopedics Department to access the entire summative assessment results of the fifth-year medical students throughout this academic year. We recruited the MCQ scores obtained from the total year records and documented them in record forms to apply in data analysis.

### Data analysis

Before performing the comparative and correlation analyses, we separated the students into two groups based on how many of the web-based illness scripts each student had completed. The cutoff point of 60% completion of the illness scripts was used since we regarded this as an acceptable response rate,^19^ which was approved by the Orthopedic Undergraduate Education Committee. The students completing 60% or more of the scripts were assigned to the high-response group, while students completing fewer than 60% were assigned to the low-response group.

The characteristics of the students, completion of the illness scripts, and examination scores in summative assessment were compared between the two groups. Categorical variables are presented in absolute numbers with percentages and compared between the two groups using the Chi-square test. Continuous variables are presented in means and standard deviations and compared between the two groups using a two-sample t-test or Mann-Whitney U-test, as appropriate. Pearson correlation coefficients (r) were used to test the correlations between the completion of the scripts, illness script scores, and examination scores. We also did a linear regression analysis to identify the relationship between illness script scores and examination scores in the high-response group. Independent variables were illness script scores from the ten orthopedic problem domains, and total scores were controlled for gender and grade point average, while the dependent variable was the examination scores from the MCQs in summative assessment. The associations are presented using regression coefficients, and statistical significance was considered at p<0.05. All data were analyzed using the R program version 4.0.3 (The R Foundation for Statistical Computing, 2020, Vienna, Austria).

## Results

### Demographics

Of the 83 fifth-year medical students in one academic year, 41 (49.4%) were female, and 42 (50.6%) were male. All were Thai and single. The average cumulative GPA was 3.3 (SD=0.32). After separation into two groups, 56 (67.47%) were in the high-response group and 27 (32.53%) were in the low-response group. When characteristics, examination scores, and completed illness scripts were compared between the two groups (Table 1), there were no significant differences in gender, cumulative grade point average, or examination scores between the two groups. However, there was a statistically significant difference in the completed illness scripts between the two groups (t_(__27)_=13.72, p<0.001).

**Table 1:** Demographic data compared between the high-response and low-response groups

Variable	high-response group (n=56) Mean ± SD	low-response group (n=27) Mean ± SD	p
Gender			0.389
Male N (%)	26 (46.4)	16 (59.3)	
FemaleN (%)	30 (53.6)	11 (40.7)	
Grade point average (0-4)	3.28 ± 0.33	3.34 ± 0.32	0.485
Completed illness scripts (%)	93.79 ± 6.59	22.72 ± 6.51	<0.001*
Examination scores (%)	64.61 ± 9.05	67.41 ± 8.35	0.169

*Statistical significance (p

### Relationship between completed illness scripts and examination scores

Negatively weak correlations without statistical significance were found between the completion of illness scripts and examination scores (r=-0.09, p=0.44).

### Relationship between illness script scores and examination scores

We found a negative, weak, and non-significant correlation between overall illness script scores and examination scores (r=-0.03, p=0.78). Specifically, we explored the relationship between illness script scores in each domain and examination scores only in the high-response group. The results of the linear regression analysis are shown in Table 2. We found no significant relationship between illness script scores in any orthopedic domain and examination scores, even in the high-response group. There was also no significant relationship between total illness script scores and examination scores after controlling for gender and grade point average (β=-1.07, p=0.143).

**Table 2:** Linear regression analysis between illness scripts scores and examination scores in the high-response group

Illness script	Regression Coefficient	Standard Error	Beta	t	p
Elbow and hand	-0.404	1.643	0.053	-0.246	**0.807**
Shoulder	-1.573	1.642	-0.241	-0.958	**0.344**
Foot and ankle	-0.536	1.267	-0.101	-0.422	**0.675**
Hip	0.235	1.194	0.052	0.197	**0.845**
Knee	0.021	1.219	0.005	0.018	**0.986**
Spine	0.820	1.348	0.165	0.608	**0.546**
Back	-0.632	1.554	-0.135	-0.406	**0.687**
Orthopedic pediatrics	-1.662	1.422	-0.320	-1.169	**0.250**
Orthopedic trauma	-0.520	0.829	-0.147	-0.628	**0.534**
Orthopedic oncology	2.562	1.329	0.503	1.927	**0.061**

## Discussion

We assumed, following various studies, that practicing clinical reasoning from illness scripts could improve the ability to organize medical knowledge and framework thinking.^6^^,^^9^ Therefore students who completed a higher number of web-based illness scripts would be more likely to show better performance in their orthopedics rotation. Contrary to our hypothesis, however, we found no positive relationship between the number of web-based illness scripts completed and the students' performance as assessed through summative examination results in their orthopedic surgery placement.

There are several possible explanations as to why the number of illness scripts completed did not correlate with the performance of the students. One explanation could be the performance assessment method. After one month of placement, we conducted summative examinations to assess the medical students' performance in relation to the learning objectives, including appropriate diagnosis, treatment principles, disease prevention, and fundamental orthopedic procedures. Most of the questions in the examination were comprehensive. In contrast, illness script questions consisted of MCQs and short answers, looking for only a diagnosis and its vital features, including the disease's signs and symptoms, epidemiology, and pathophysiology, following a previous case-based illness script pattern.^12^ Inferring from the study results, the medical students who completed more illness scripts for diagnosis may have had more potential to integrate the newly learned knowledge into their existing fund of knowledge, leading to obtaining higher scores in the comprehensive questions seeking a correct diagnosis. Nevertheless, they may not have obtained high total MCQ examination scores since they could not apply the stored knowledge acquired from the illness scripts for diagnosis to choose the appropriate answer for treatment or management. Moreover, non-diversified type of questions for evaluation was suspicious for non-significant results. In our research, we compared the results by calculating only MCQ scores. The MCQ is one of the preferred assessment tools for evaluating undergraduate medical students,^20^ and although assessment with MCQs is reliable, various types of theoretical examinations for evaluating students' knowledge and performance should be utilized.^21^^,^^22^ Our results may have been different if other methods of assessment had been included in the study.

Another possible factor that may have impacted our results was the large number of online illness scripts which expanded the students' workloads.^23^^,^^24^ We intended to provide scripts thoroughly covering all common orthopedic problem domains, and created a total of 74 scenarios. The students needed to spend a significant amount of time working on each script while at the same time learning from the answer page. However, they had only one month in the orthopedic placement, and doing all of the online illness scripts may have reduced the time available to prepare for the summative examination. Because of the time limitations, the students may have paid more attention to the examination to attain high scores, enhancing their cumulative GPA and perhaps influencing future careers.^25^ As a result, some students may have gained high examination scores despite being in the low-response group.

Additionally, online learning courses have their own limitations, as reported in previous studies,^14^^,^^26^ which identified various problems or barriers such as skill deficits in practice, lack of technology for reaching the online course, a negative attitude amongst instructors, or learner motivation. We suspect that the personal learner motivation factor may have influenced the number of completed illness scripts assigned as optional and thus leading to our expectations not being met.

Although our study failed to confirm that completing our online illness scripts would increase the summative examination scores, we still believe that studying online illness scripts can be very useful in helping the students develop and organize the knowledge required for skilful disease diagnosis, including understanding which further investigations may be required, and treatments, all of which are essential for providing proper management for their patients.^9^^,^^13^ The benefits from illness scripts are realized more in medical students who have the dedication to practice and learn from modern instruction tools such as web-based scripts.^4^ We learned from the first use of this teaching technique that some modifications of web-based illness scripts in orthopedics are required, especially by considering an appropriate number of illness scripts, adding some scripts about orthopedic disease management, deleting some scripts about diagnoses, and combining the scripts with face-to-face teaching.^27^^,^^28^

There were some limitations to our study. This research was a retrospective study which has inherent weaknesses. Some important data were not recorded, and we could not control all of the baseline characteristics and attitudes of students that may be confounding factors, but this was unavoidable due to the study design. Moreover, this was conducted in a single-center academic hospital with a small number of medical students. Thus, we did not have much information in our online database system for analysis. Another limitation was that we studied the relationship of illness scripts and performance in fifth-year students in an orthopedic surgery placement only; hence, the generalization of the results in this study to other departments may be limited. Lastly, we used a fixed cut-off point without randomized allocation to separate the high-response and low-response groups, leading to possible selection bias and internal validity inconsistencies.

## Conclusions

In this study, the use of web-based illness scripts in orthopedics was not correlated with the medical students' performances, as assessed through examination results, following a one-month placement. We identified several factors which potentially obscured the benefits of the web-based illness scripts. For modifiable factors, a perhaps too high number of illness scripts only in diagnosis and inadequate time for practice in a short one-month rotation may have negatively affected the performance assessment of the medical students in the study. These factors should be considered and adjusted in the proper context for each medical school when creating illness scripts and assigning them as tasks.

A further multi-center, prospective study with a larger sample size from more than one academic year is suggested to identify the relationship between web-based illness scripts in orthopedics and the students' performance. It is also recommended to investigate the factors that positively or negatively affect this relationship in future studies. The strengths and weaknesses of web-based illness scripts in orthopedics are still uncertain.

### Acknowledgements

We would like to thank Assoc. Prof. Boonsin Tangtrakulwanich of the Orthopedics Department for his contribution in revision and editing, and Mr. Dave Patterson of the International Affairs Office of the Faculty of Medicine, Prince of Songkla University, for his assistance in proofreading the English of this study. We would also like to express our appreciation to the undergraduate medical education team in the Orthopedics Department for their assistance.

### Conflicts of Interest

The authors declare that they have no conflict of interest.
